# Supplementary material for: Controlling the Treatment Time for Ideal Morphology towards Efficient Organic Solar Cells
Source: Molecules. 2022 Sep 5;27(17):5713. doi: 10.3390/molecules27175713 (PMC9457995; doi:10.3390/molecules27175713)
Supplement: Supplementary file 1 [file molecules-27-05713-s001.zip › molecules-1895580-supplementary.pdf]

Supporting Information:

Controlling the Treatment Time for Ideal Morphology Towards Efficient Organic Solar Cells

Table S1. Independent device results.

PM6:SY1	V_{OC} (V)	J_{SC} (mA cm^{-2})	*FF*	PCE (%)
SVA 0s				
	0.86232	25.40275	0.76617	16.78326
	0.86054	25.29988	0.75712	16.48362
	0.86107	25.27381	0.75963	16.53149
	0.86006	25.39	0.74973	16.37115
	0.8691	25.13252	0.77357	16.89696
	0.86535	24.83365	0.76179	16.37074
	0.8632	24.66915	0.75714	16.12275
	0.86505	25.73804	0.75691	16.8523
	0.85954	25.33685	0.69967	15.23743
	0.86229	25.47181	0.75006	16.47433
	0.8617	25.31363	0.74445	16.2386
	0.86214	25.06508	0.75777	16.37067
	0.8663	25.06925	0.75526	16.39054
	0.86389	25.35031	0.75757	16.5906
	0.86231	25.03085	0.75991	16.40196
	0.86179	25.6489	0.74667	16.50426
	0.86357	25.54983	0.75235	16.59986
	0.85517	25.62077	0.75724	16.58855
	0.86503	25.49379	0.75756	16.69219
	0.86043	25.16645	0.75484	16.34349
SVA 30s				
	0.87232	25.94175	0.76656	17.34676
	0.87054	25.79098	0.75896	17.04026
	0.87107	25.80325	0.76034	17.08974
	0.86633	26.05182	0.76588	17.28553
	0.87006	25.92195	0.75043	16.92396
	0.86389	25.86432	0.77569	17.33192
	0.86535	25.30333	0.77927	17.05061
	0.8632	25.83212	0.76949	17.15825
	0.86497	26.27337	0.76762	17.44412
	0.87029	25.81012	0.77063	17.31027
	0.86705	25.8021	0.7765	17.37164
	0.87014	25.97557	0.76911	17.38389
	0.8763	26.14804	0.75772	17.36211

	0.86389	25.82984	0.76967	17.17448
	0.86931	26.01743	0.77216	17.46429
	0.86179	26.10684	0.77411	17.41396
	0.86357	26.02554	0.7748	17.41364
	0.8691	26.13782	0.77357	17.57284
	0.86232	25.91886	0.77617	17.34772
	0.86032	25.88623	0.77717	17.30155
SVA 50s				
	0.8579	25.64998	0.74056	16.29619
	0.85643	26.11	0.73468	16.42845
	0.85517	25.62077	0.75724	16.58855
	0.85348	25.93532	0.73919	16.3523
	0.85574	26.07902	0.73235	16.34276
	0.86643	26.16645	0.73484	16.65983
	0.86574	26.10636	0.73329	16.57215
	0.86232	25.40275	0.76617	16.78326
	0.86054	25.99885	0.75712	16.93562
	0.86107	25.27381	0.75963	16.53149
	0.86006	25.99	0.74973	16.75615
	0.86535	25.33654	0.76179	16.70041
	0.8632	25.66915	0.75714	16.76952
	0.8617	25.97163	0.75036	16.7976
	0.86214	25.46508	0.73777	16.20067
	0.8663	25.66925	0.75526	16.79496
	0.86389	25.35031	0.75757	16.5906
	0.86231	25.53085	0.74991	16.50962
	0.86179	25.6489	0.74667	16.50426
	0.86357	25.54983	0.75235	16.59986

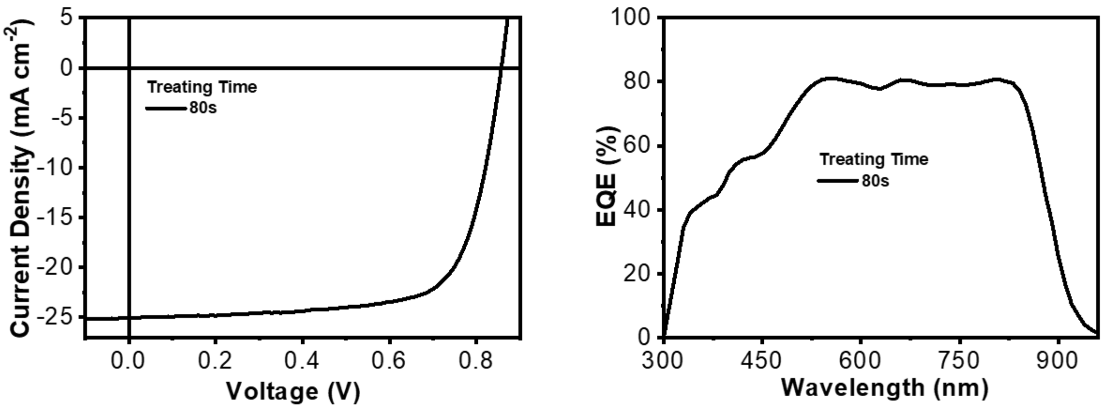

Figure S1. *J-V* characteristics and EQE spectra for SVA 80s device.

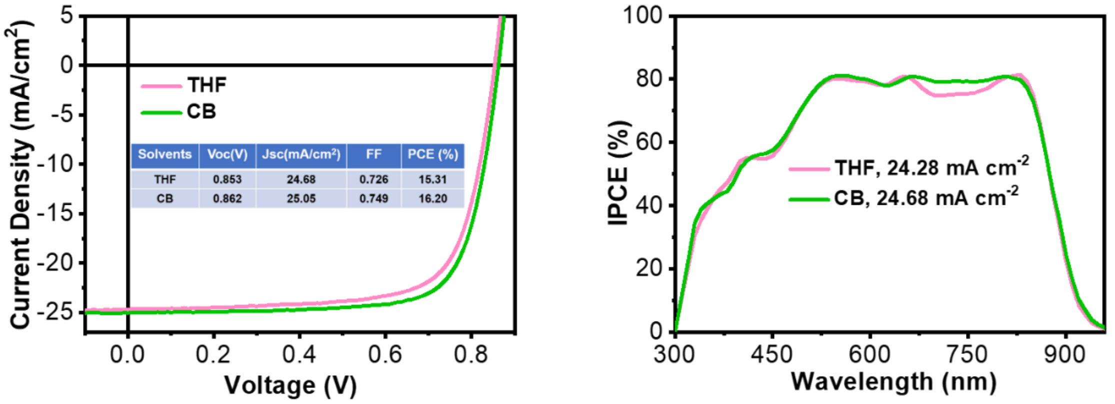

Figure S2. *J-V* characteristics and EQE spectra for THF and CB treated devices.
